# The Effects of Chia Defatted Flour as a Nutritional Supplement in C57BL/6 Mice Fed a Low-Quality Diet

**DOI:** 10.3390/foods13050678

**Published:** 2024-02-23

**Authors:** Agustin Lucini Mas, Alejandra Mariel Canalis, María Eugenia Pasqualini, Daniel Alberto Wunderlin, María Verónica Baroni

**Affiliations:** 1 Instituto de Ciencia y Tecnología de Alimentos Córdoba (ICYTAC-CONICET), SeCyT—Universidad Nacional de Córdoba, Córdoba X5000GYA, Argentina; agustin.lucini@unc.edu.ar (A.L.M.); amcanalis@fcm.unc.edu.ar (A.M.C.); daniel.wunderlin@unc.edu.ar (D.A.W.); 2 Departamento de Química Orgánica, Facultad de Ciencias Químicas, Universidad Nacional de Córdoba, Córdoba X5000GYA, Argentina; 3 Instituto de Investigaciones en Ciencias de la Salud (INICSA-CONICET), Pabellón Biología Celular, Universidad Nacional de Córdoba, Córdoba X5000GYA, Argentina; eugepasqualini@gmail.com; 4 Escuela de Nutrición, Facultad de Ciencias Médicas, Universidad Nacional de Córdoba, Córdoba X5000GYA, Argentina; 5 Instituto de Biología Celular (IBC-UNC), Cátedra de Biología Celular, Histología y Embriología, Facultad de Ciencias Médicas, Universidad Nacional de Córdoba, Córdoba X5000GYA, Argentina

**Keywords:** oxidative stress, chia, glucose metabolism, polyphenols, antioxidant, high fructose, high saturated fatty acids

## Abstract

Today, consumption of diets rich in saturated fat and fructose, associated with a variety of metabolic deregulations, has increased. The aim of this study was to evaluate the effect of dietary supplementation with a residue of defatted chia seed on a diet with low nutritional quality. To do this, C57BL/6 male mice were fed with the Control (C), Low-Nutritional-Quality (LNQ), or supplemented-with-chia-defatted-flour (LNQ+C) diets. After 12 weeks, the glucose and lactate levels were determined in the serum, liver, and kidney, along with reactive oxygen species (ROS) levels, antioxidant enzyme activity, reduced glutathione (GSH), and protein oxidation (AOPP). The LNQ diet increased the glucose and lactate levels (+25% and +50% approx. in the liver, with respect to the control group) and generated oxidative stress by modifying the levels of ROS and the activity of antioxidant enzymes, causing oxidative damage to proteins (+12% in the liver, with respect to the control). Chia supplementation helped to restore the glucose to control levels and modulate the endogenous antioxidant system, resulting in a decrease in protein oxidation products with no differences compared to the control group. In conclusion, supplementation with chia showed beneficial effects on the general health of mice, even when fed a low-nutritional-quality diet.

## 1. Introduction

Today, consumption of high-saturated-fat and high-sugar diets has notably increased due to a busy lifestyle that leads to the intake of ultra-processed food and due to the greater availability of low-nutritional-value products on the market.

It is well known that a high-fat diet could generate dyslipidemia with high plasma concentrations of triglycerides and low concentrations of high-density lipoprotein cholesterol [1]. In addition, this type of deregulation is not only linked to high-fat diets but also to high consumption of saturated fatty acids. Likewise, high sugar intakes or, specifically, high fructose diets have effects on the body metabolism as well. Fructose is more lipogenic than sucrose and could generate liver steatosis, increasing blood triglycerides; it may also develop insulin resistance, leading to increased fasting glucose levels [2]. All these metabolic deregulations are also associated with an increased production of reactive oxygen species, causing oxidative stress, which can damage different macromolecules. These dietary patterns are linked to the development of certain metabolic deregulations and diseases such as type II diabetes mellitus, atherosclerosis, hypertension, and certain types of cancer such as breast, endometrial, prostate, gastric, and pancreatic cancer [3].

To develop a proper and healthier nutrition, it is recommended to change dietary patterns and incorporate regular physical activity. However, modifying dietary practices not only implies a reduction in fat and sugar but also supplementation with bioactive compounds. It is well known that the consumption of polyphenolic compounds helps reduce oxidative stress and could also modulate different pathways related to lipid and carbohydrate metabolism [4]. Polyphenols are plant-derived organic compounds that contain phenolic rings with at least one or more hydroxyl that can be substituted with different alkyl groups. Its antioxidant activity or ability to modulate different metabolic pathways depends not only on the concentration consumed but on the structure of each compound.

Chia seeds are rich in fiber, polyunsaturated fatty acids, and antioxidant compounds [5]. Hence, these seeds are being studied on different pathologies associated with nutrition. Oliva et al. [6] found that supplementation with whole chia seeds helped reduce visceral fat accumulation and improve insulin sensitivity and plasma lipid profile in male Wistar rats when fed with a sucrose-rich diet. Enes et al. [7] also showed that different seed portions had different effects on glucose metabolism in male Wistar rats fed with high fat and high fructose diets, since both chia flour and oil reduced adiposity and increased AMPK mRNA. In the same study, it was demonstrated that a phenolic extract from these seeds increased the gene expression of gluconeogenic and glycolytic enzymes on HepG2 insulin resistant cells. In addition, da Silva Marineli et al. [8] also studied the antioxidant effect of chia seeds by supplementing a high-fat/high-fructose diet with chia seeds or oil. They found that both chia fractions increased the reduced thiol, modulated antioxidant enzymes, and reduced lipid peroxidation. Alarcon et al. [9] demonstrated that chia defatted residue supplementation improved the fasting glucose, insulin resistance, and the triglycerides/glucose index in rabbits fed a high-fructose-high-fat diet. Finally, Duarte Villas Mishima et al. [10] showed that chia supplementation improved the intestinal morphology and functionality in young Wistar rats fed with a high-fat diet.

For all these beneficial effects, chia oil is extracted to be marketed as a food additive or dietary supplement. From this process, a byproduct rich in polyphenolic compounds is obtained [11]. Thus, the use of this byproduct would not only show beneficial effects on consumer’s health, but it would also help reduce waste from the food industry, largely contributing to environmental sustainability [12].

To our knowledge, there are no published studies with an integrated approach that use an industrial byproduct of chia seeds as a supplement to improve the effects of a poor quality diet. Therefore, the aim of this study was to evaluate the effect of this residue of defatted chia seeds on the metabolism of carbohydrates and the general redox state of mice when fed with a low nutritional value diet (high saturated fat and high fructose). The diet used in this study emerged from an epidemiological study carried out in the city of Córdoba, Argentina, where real dietary patterns were identified [13].

## 2. Materials and Methods

### 2.1. Chemicals and Reagents

Ultra-pure water (<18 MΩ·cm, <5 μgL^−1^ TOC) was obtained from a purification system Arium 61316-RO plus Arium 611 UV (Sartorius, Goettingen, Germany). Nitroblue Tetrazolium, Xylenol Orange, Glutathione Reductase, Reduced Glutathione, Oxidized Glutathione, NADPH, and Ellman’s reagent and glucose determination commercial kit were provided by Sigma Aldrich (Buenos Aires, Argentina). The lactate determination commercial kit was obtained from Wiener Lab Group (Buenos Aires, Argentina) and Isoflurane from Piramal Healthcare (Morphet, UK). All other reagents were of analytical grade.

### 2.2. Diet Preparation

Three different diets were prepared to evaluate the effect of chia defatted flour as a nutritional supplement in the carbohydrate metabolism and general redox state of mice.

First, a control diet (C) with all animals’ nutritional needs was prepared following AIN-93M specifications [14]. Second, a low-nutritional-quality diet (LNQ) was prepared according to the dietary patterns determined in the epidemiological study described by Pou et al. [13]. In this diet, no fiber was present, sucrose was totally replaced with fructose, and corn oil (rich in unsaturated fatty acids) was replaced with palm oil (rich in saturated fatty acids). In addition, the percentage of calories from each macronutrient varied from 15% protein, 68% carbohydrates, 4% fiber, and 13% fat in the control diet to 17% protein, 66% carbohydrates, and 17% fat in the Low-Nutritional-Quality diet. Finally, a third diet was prepared replacing 10% of weight of the LNQ with chia defatted flour (LNQ+C) obtained according to Lucini Mas et al. [11]. In this diet, chia defatted flour provided 2.77% protein, 4.13% fiber, and 1.36% lipids (rich in polyunsaturated fatty acids) that were adjusted from the other components to obtain the same percentage of calories from each macronutrient as those in LNQ. This diet also contained 383.73 µg of polyphenols from chia defatted flour per g of diet, which were identified and quantified using HPLC-MS/MS according to Lucini Mas et al. [11]. The percentage of chia substitution was based on the previous literature [7,8]. In addition, a larger replacement would increase the fiber composition far above the control diet, which is considered the best nutritional composition for these animals.

The content of the diets is described in Table 1. In addition, the chia defatted flour composition and the polyphenol identification and quantification are summarized in Tables S1–S3 of the Supplementary Materials.

### 2.3. Animals and Experimental Design

Twenty four male C57BL/6 mice of 3 weeks of age with homogenous body weights (15.2 ± 2.5) were obtained from the Mercedes and Martin Ferreyra Medical Research Institute (CONICET, Córdoba, Argentina). The animals were placed under standard controlled conditions (21 °C ± 1 °C; 12 h day/night cycle) and randomly assorted into three different experimental groups (*n* = 8), according to the diet administered (C, LNQ, or LNQ+C). Food and water were provided ad libitum, and their consumption was monitored weekly along with their general status and body weight, following the experimental conditions of our previous studies [15]. After 12 weeks of being fed on the specific diets, the animals were fasted for 8 h, completely anesthetized by isoflurane inhalation, and then euthanized by exsanguination, according to the accepted experimental procedures. Blood samples were obtained by cardiac puncture and then centrifuged at 3000 rpm for 10 min, in order to obtain the serum. Subsequently, in the autopsy of each individual, the livers and kidneys were immediately removed. The organs were weighed, frozen in liquid nitrogen, and stored at −80 °C until sample preparation. At the end of the experiment, body weight gain was calculated as

$$\frac{(\mathrm{Final}\;\mathrm{Weight}\;-\;\mathrm{Initial}\;\mathrm{Weight})}{\mathrm{Initial}\;\mathrm{Weight}}\times 100$$

All the experimental procedures complied with the Guide for the Care and Use of Laboratory Animals issued by the National Institutes of Health [16]. The experimental protocol was approved by the Institutional Committee for the Care and Use of Laboratory Animals (Comité Institucional para el Cuidado y Uso de Animales de Laboratorio (CICUAL)) of the Mercedes and Martin Ferreyra Medical Research Institute, CONICET, Córdoba, Argentina (ACTA 010/2021A).

### 2.4. Metabolism of Carbohydrates: Determination of Glucose and Lactate Concentration

Glucose and lactate were measured as they are intermediaries of the aerobic and anaerobic metabolism of carbohydrates, respectively [17].

In order to perform these determinations, an aliquot of liver or kidney tissue was mechanically homogenized in saline isotonic in 5% g_tissue_/mL_buffer_ proportion using an UltraTurrax homogenizer. The homogenates were then centrifuged at 10,000× *g* at 4 °C for 10 min, and the supernatants were collected and used for the determinations [15]. The remaining aliquot of the supernatants was collected, frozen in liquid nitrogen, and stored at −80 °C until further analysis.

The glucose concentration was determined in serum (without any preparation) and liver and kidney homogenates at 505 nm using a commercial kit based on the formation of quinoline in the reaction of H_2_O_2_, formed from the oxidation of glucose present in samples with 4-aminophenazone and phenol. The results were expressed as μg_glucose_/mg_protein_. The protein concentration of the samples was determined by the Bradford method, based on the color change of the Coomassie brilliant blue G-250 colorant when bound with the basic amino acid residues, which can be measured spectrophotometrically at 595 nm [18]. 

Lactate concentration was also measured in the serum and in liver and kidney homogenates using a commercial kit based on its reaction with enzyme lactate oxidase that generates H_2_O_2_, forming a complex from the action of peroxidase. The absorbance of this complex at 540–550 nm is proportional to the lactate concentration. The absorbance was standardized by the protein concentration and expressed as percentage of the mean of samples of the animals fed the control diet.

### 2.5. Analysis of General Redox State of Animals

#### 2.5.1. Reactive Oxygen Species

##### Superoxide Determination

The determination of O_2_^●−^ was performed using nitroblue tetrazolium chloride (NBT) in a 1 mg/mL concentration. NBT reacts with O_2_^●−^ to form a dark purple/blue formazan precipitate, which can be quantified using a spectrophotometer after being dissolved [15].

Briefly, the liver and kidney homogenates (Section 2.4) and serum were mixed with the colorant in a proportion of 9:1 *v*:*v* and incubated for 30 min in darkness at 37 °C. Then, DMSO and an aqueous solution of NaOH 2 M were added to the mixture (1:1:2 *v*:*v*). Finally, the absorbance of the resulting solution was measured in triplicate at 600 nm. The O_2_^●−^ concentration was calculated as the percentage of the mean of the absorbance of the samples from animals fed the control diet, after being standardized by the protein concentration as described previously.

##### Hydroperoxide Determination

The concentration of aqueous hydroperoxides (AHP) and lipid hydroperoxides (LHP) was measured in the serum and in the liver and kidney homogenates (Section 2.4), by their ability to react with the colorant xylenol orange [15].

In the measurement of the AHP, the samples were mixed in a proportion of 1:10 *v*:*v* with the colorant solution for 30 min at room temperature. The colorant solution consisted of ferrous ammonium sulfate 25 mM, sorbitol 100 mM, and xylenol orange 125 µM in sulfuric acid 2.5 M. 

In the case of LHP, the same procedure was followed; however, the colorant solution was prepared using ferrous ammonium sulfate 25 mM, butylated hydroxytoluene 4 mM, and xylenol orange 125 µM in methanol 90%.

Both the AHP and LHP were measured in triplicate at 500 nm and expressed as percentages of the mean of the absorbance of samples from animals fed the control diet, after standardizing by protein concentration.

#### 2.5.2. Analysis of the Antioxidant Enzyme Activity

The activity of the antioxidant enzymes catalase (CAT), glutathione peroxidase (GPx) and glutathione reductase (GR) was measured in the liver, kidney, and serum. In order to carry out these determinations, another tissue homogenate was obtained. Briefly, an aliquot of liver or kidney tissue was homogenized using ice-cold 0.1 M potassium phosphate buffer, pH 6.5, containing 20% (*v*/*v*) glycerol, 1 mM EDTA (ethylenediaminetetraacetic acid), and 1.4 mM dithioerythritol (DTE) in a proportion of 5% g tissue/mL buffer. Then, the cell debris was removed by 10 min of centrifugation at 13,000× *g* and 4 °C using a refrigerated ultracentrifuge (Sorvall Ultraspeed). The supernatant was then collected.

The catalase activity was determined by following the decrease in absorbance at 240 nM of the substrate H_2_O_2_ when mixed with the sample properly diluted in the phosphate buffer 50 mM, pH = 7 [19]. 

The determination the activity of glutathione peroxidase was based on the oxidation of glutathione by the GPx present in the sample (properly diluted with phosphate buffer 0.1 M, pH = 7.5), using H_2_O_2_ as a substrate, coupled with the disappearance of NADPH by the commercial glutathione reductase added to the mixture [20].

Finally, the activity of glutathione reductase was measured directly by the disappearance of NADPH when mixed with GSSG and the sample diluted in the same buffer used for GPx [21]. The disappearance of NADPH for the last two methods was measured at 340 nm.

All enzymatic activities were measured in triplicate and reported in nano katals per milligram of protein (nkat/mg_prot_), where 1 kat is the conversion of 1 mol of substrate per second. 

#### 2.5.3. Determination of Free Thiol Groups (Reduced Glutathione)

To determine the level of non-enzymatic endogenous antioxidant in serum and tissue homogenates (Section 2.4), free thiol groups (-SH) were measured using Ellman’s reagent. The concentration of free thiol was correlated to reduced glutathione (GSH), as it is one of the major free thiols in the organism.

Ellman’s reagent (DTNB or 5,5′-dithiobis-(2-nitrobenzoic acid)) reacted with thiol groups in alkaline conditions to form the yellow product 2-nitro-5-thiobenzoic acid (NTB) that could be measured at 412 nm [22]. The concentration of thiol groups was expressed as mg_SH_/mg_prot_ calculated by extrapolating from a standard curve made with n-acetylcysteine and standardized by the protein concentration.

#### 2.5.4. Determination of Advanced Oxidation Protein Products

The oxidative damage in proteins was measured through the oxidation of proteins by the advanced oxidation protein product method (AOPP) [23]. In acid conditions, AOPP could react with iodide anion to form molecular iodine, and this disappearance could be measured at 340 nm. Briefly, an aliquot of tissue homogenate sample (Section 2.4) was mixed with PBS 0.015 M, IK 1.16 M, and acetic acid in a 4:16:1:2 proportion. The AOPP concentration was estimated as mg_chloramineT_/mg_prot_ using a calibration curve created with chloramine T as standard and then normalized by the protein concentration.

### 2.6. Statistical Analysis

For statistical analysis, the software INFOSTAT 2020 version was used [24]. All results were expressed as percentage of control samples to improve visualization. Normality and homoscedasticity were evaluated graphically and numerically (using tools of R library fitdistrplus) through Shapiro–Wilk’s tests. General and Linear Mixed Models were used to evaluate significant differences in the effect of the diet. This method assumes that although the response variable is linearly related to a fixed factor that models the mean value, there are random effects that must be included in the lineal predictor. In our study, the diet supplied was included as a fixed effect, and the experiment repetition was included as a random effect. In the case of significance (*p* < 0.05), an LSD Fisher comparison test was performed to reveal paired differences between the means. Data are expressed as mean ± SE. 

## 3. Results

### 3.1. Effects of Diets on Body Weight and Food and Water Intake

After 12 weeks of experimental conditions in which the mice were fed three different diets (Control, Low-Nutritional-Quality, and Low-Nutritional-Quality supplemented with 10% of Chia defatted flour), no changes were observed in the coat or coloration of the skin and mucous membranes. The results of the effects of diets on body weight and food and water intake are shown in Table 2. The measurement of the body weight gain showed no significant differences between the experimental groups. However, the LNQ+C followed an upward trend (0.05 < *p* < 0.1) in relation to the other two diets. The water intake and weight of the organs studied in this work showed no changes between the groups. 

### 3.2. Effects of Diets on the Aerobic and Anaerobic Metabolism of Carbohydrates

**Figure 1:**
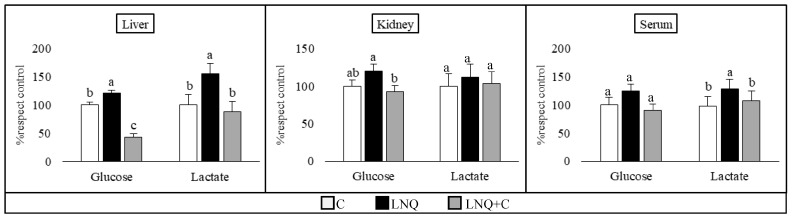
Glucose and lactate in liver, kidney, and serum. (*n* = 8 per group). The results are expressed as the mean ± SE of percentages with respect to the control diet. Different letters indicate significant differences (*p* < 0.05) between the different diet groups for the same parameter in the same tissue.

The glucose and lactate levels in the liver, kidney, and serum are expressed in Figure 1. The liver samples showed an increase in both the glucose and lactate concentration when animals were fed with LNQ, compared to the control group. However, supplementation with chia helped decrease both, even under the control level in the case of glucose. In the case of the kidney, only the glucose level showed significant differences between the LNQ and LNQ+C groups, while the lactate levels showed no modifications. Finally, the serum samples revealed differences in the lactate concentration, with the LNQ group having the highest values, while the LNQ+C helped decrease it to the control levels. The glucose levels had no significant differences in these samples.

### 3.3. Effects of the Nutritional Quality of the Diets in the General Redox State

#### 3.3.1. Levels of Reactive Oxygen Species

**Figure 2:**
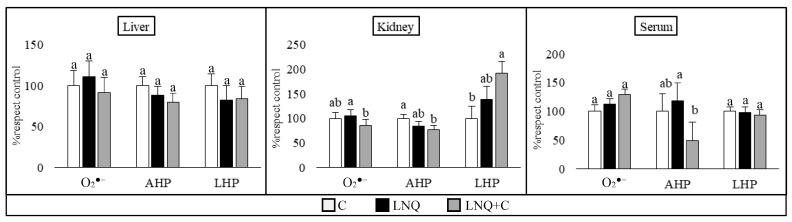
Reactive oxygen species in liver, kidney, and serum. (*n* = 8 per group). The results are expressed as the mean ± SE of the percentages with respect to the control diet. Different letters indicate significant differences (*p* < 0.05) between the different diet groups for the same parameter in the same tissue.

The results of the determination of ROS (superoxide (O_2_^●−^), aqueous hydroperoxides (AHP), and lipidic hydroperoxides (LHP)) are summarized in Figure 2.

In the liver samples, no significant differences were observed in the ROS. However, in the kidney tissue, O_2_^●−^ was increased in the LNQ group, while supplementation with chia defatted flour helped decrease it. Concerning hydroperoxides, the LNQ+C group showed the lowest concentration of AHP differing from those of the control group. On the other hand, the LNQ+C group had higher levels of LHP in relation to the control group, with an intermediate trend in the case of the LNQ group. Regarding the serum samples, the LNQ+C group showed the lowest AHP content.

#### 3.3.2. Antioxidant Enzyme Activity

**Figure 3:**
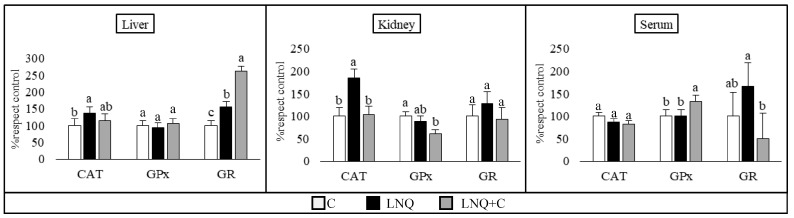
Antioxidant enzymes activities in the liver, kidney, and serum. (*n* = 8 per group). The results are expressed as the mean ± SE of percentages with respect to the control diet. Different letters indicate significant differences (*p* < 0.05) between the different diet groups for the same parameter in the same tissue.

Figure 3 summarizes the results of the activity of the three antioxidant enzymes (CAT, GPx, and GR). Regarding the liver tissue, the LNQ group had increased CAT activity; however, supplementation with chia showed a downward trend. Yet, the opposite effect was observed in the GR, where the LNQ+C group increased its activity even above the LNQ group. In kidney samples, as well as in liver ones, the LNQ group had increased CAT activity, but the LNQ+C group decreased to the control levels. On the other hand, the supplemented diet showed a reduction in the activity in GPx in relation to the control group. No significant differences were observed in the GR. Finally, the CAT showed no differences in the serum samples between diets. However, the LNQ+C group increased the GPx activity compared to the other groups, while it decreased the GR.

#### 3.3.3. Reduced Glutathione

The results of the GSH levels only showed significant differences in the liver samples, where the LNQ+C group decreased the amount of reduced glutathione in relation to the LNQ group, as shown in Table 3.

#### 3.3.4. Advanced Oxidation of Protein Products (AOPP)

The kidney tissue showed no significant differences in the AOPP between diets (Table 3), but the AOPP increased in the liver when the animals were fed the LNQ diet, compared to those on the control diet. The supplementation of the LNQ diet with chia defatted flour helped to decrease the AOPP levels. 

## 4. Discussion

Fructose and saturated fatty acid consumption is usually associated with metabolic deregulations [1,2]. In this study, no differences in body weight gain or in organ weights were observed between the diets. Despite this, an upward trend was found in the body weight gain of the LNQ+C group. Similar results were reported by Barbosa et al. [25] in which no difference in body weight was observed between groups of female Fischer rats fed a control or high-fat diet; yet, supplementation of a high-fat diet with açaí increased the body weight of animals. In our study, this effect could result from the fact that the food intake was higher for the LNQ+C group. Shen et al. [26] also found a higher food intake when a high-fat diet was supplemented with purple rice. Food consumption is determined by appetite, which in turn depends on factors such as the gastric emptying process and hormone responses. Polyphenol compounds could modulate leptin hormone production or transportation; thus, the appetite in the LNQ+C group could be affected. In addition, since all the diets comprised different nutrients with different energy density, the gastric emptying process could be conditioned [27]. Accordingly, a higher solid fat content in the LNQ group would condition a greater power of satiety mediated by the caloric content of the diet [28]. 

The diet composition not only impacts the food intake but also can have an important effect on the metabolism of carbohydrates. Numerous studies have suggested that diets with a high proportion of saturated fatty acids and fructose could cause liver steatosis that leads to insulin resistance [2,29]. These two circumstances often increase glucose and glucogenic substrates such as lactate; therefore, this could be one of the reasons why we found higher levels of both glucose and lactate in our LNQ samples. However, it is known that some bioactive compounds like polyphenols could have effects at different levels such as interaction with cell-signaling pathways, modulation of the activity of transcription factors, and consequently, the expression of genes related to inflammation, insulin resistance, and diabetes [4]. In addition, polyphenols could modulate the activity of carbohydrate digestive enzymes. In fact, Jayanthy et al. [30] found that rosmarinic acid (one of the major compounds of chia defatted flour) exerts effects on the activity of key carbohydrate-metabolizing enzymes such as hexokinase, pyruvate kinase, glucose-6- phosphatase, fructose 1,6-bisphosphatase, glucose-6-phosphate dehydrogenase, glycogen synthase, and glycogen phosphorylase in the liver, resulting in a decrease in the blood glucose level. In this way, the decrease in glucose levels in the liver and kidney and lactate in the liver and serum observed in this study may be attributed to the polyphenolic content of the LNQ+C diet.

Changes in the metabolism of carbohydrates could affect the redox balance since superoxide anion (and thus other reactive oxygen species derived from it) is generated as a byproduct of the respiratory chain [31]. Reactive oxygen species could be produced excessively due to changes in the metabolism, and this usually derives in an oxidative stress condition. If the endogenous antioxidant system cannot cope with this, damage can occur to macromolecules that are components of the cell, associated with the appearance of different pathologies [32]. In this study, different results were obtained in the liver, kidney, and serum. These tissues have different metabolism functions, and it is expected that diets impact them differently. Feillet-Coudray et al. [33] also found differences in superoxide anions between different tissues of male Wistar rats when fed a high-fat and high-sucrose diet. They found higher amounts of this ROS as compared to their control diet in the heart and muscle, but no significant differences were seen in the liver. In general, polyphenol supplementation has an antioxidant effect since it helps decrease ROS levels, like in the case of O_2_^●−^ in the kidney and AHP in the kidney and serum. Canalis et al. [15] also reported lower levels of AHP in the blood samples of male BALB/c mice when treated with *Lantana grisebachii* extracts. However, in some cases, polyphenols could increase the ROS production acting as mild pro-oxidants. Cittadini et al. [34] found increased levels of LHP in different encephalic regions of female BALB/c mice when fed with water extracts of *Aspidosperma quebracho-blanco*, *Lantana grisebachii*, and *Ilex paraguariensis*. The same effect was observed in this study in LHP in the kidney. 

In the same way, enzyme activity does not follow linear responses to different stimuli, since it depends on the enzyme itself, the intensity and duration of the stressful stimulus, or the structure of the bioactive compound and its concentration. Sometimes it is expected that a moderate oxidative stress activates antioxidant enzymes and increases their activity to cope with it, while the consumption of exogenous antioxidants like polyphenols helps revert this situation. Barbosa et al. [25] found that CAT, GPx, and SOD (superoxide dismutase) activities increased in Fisher female rats fed a high-fat diet, while supplementation with açaí helped to decrease it. Wang et al. [35] observed that Caco-2 cells stressed with tert-butylhydroperoxide increased the GPx and GR activities, but grape phenolic extract, gallic acid, or syringic acid helped to decrease it. This effect may be occurring in the case of the CAT in the liver and kidney and the GR in the serum. On the other hand, some authors suggest that polyphenols may have a slightly pro-oxidant effect and thus prepare the body for greater oxidative stress. Park et al. [36] found that mouse serum SOD and GPx activities did not change when animals were fed with a high-fat and high-fructose (HF-HF) diet, compared to a control one; however, when this HF-HF diet was supplemented with black ginseng, both enzyme activities increased. This could account for what was observed in this study in the GR in the liver and the GPx in the serum. 

According to the activity of antioxidant enzymes, it would be expected that the LNQ+C group would have higher levels of GSH, since GR was highly activated. The method used to estimate the GSH levels uses a DTNB reagent that could react with all free thiol groups present in the tissue. Yorimitsu et al. [37] found that oxidative stress could cause protein misfolding, which led to protein autophagy. This could increase the levels of precursor metabolites such as S-adenosylmethionine and cysteine and, therefore, increase this method signal, showing a higher content in LNQ samples. Furthermore, glutathione could be synthetized de novo when cells require it, due to the oxidative stress to which they are subject, as in the LNQ group. Cho et al. [38] found that in endothelial cells under oxidative stress, oxidized-LDL stimulated the expression of γ-glutamylcysteine synthetase (γ-GCS), the rate-limiting enzyme for GSH synthesis, which increased the GSH concentration. Finally, it is known that insulin can stimulate GSH synthesis; thus, an imbalance in insulin levels due to a diet rich in fructose and saturated fatty acids or due to the effects of polyphenols in insulin resistance may affect this endogenous antioxidant. Cai et al. [39] found higher γ-glutamylcysteine synthetase mARN levels and the highest GSH content when hepatocytes isolated from male Sprague–Dawley rats were treated with insulin. Altogether, these could explain the higher levels of GSH in the LNQ group than in the LNQ+C group.

Even when changes in diet composition differently affected the ROS production and the activity or composition of the endogenous antioxidant system, the AOPP acted as a marker of oxidative damage suffered by the organism. In this study, the LNQ diet caused higher protein damage compared to C with significant differences only in the liver, even when the ROS did not show any significant differences in this tissue (probably due to a low-sensitivity technique). Sabir et al. [40] found elevated AOPP levels in the liver of male Sprague–Dawley rats fed a high-fat diet, correlated with an increase in the reactive nitrogen species. Hence, the protein damage observed in this study could also be produced by other mechanisms apart from the ROS. On the other hand, we found that supplementation of LNQ with chia defatted flour helped to decrease the AOPP levels. The same results were reported by Feillet-Coudray et al. [33] when they supplemented a high-fat high-sucrose rat diet with a wine polyphenol extract. Miah et al. [41] also found that supplementation of a high-fat diet with cumin seed powder decreased the AOPP levels in rats.

On the other hand, some authors attributed metabolic deregulations and oxidative stress to changes in the intestinal microbiota composition caused by high-fat or high-fructose diets. Thus, ameliorative effects could be related to the prebiotic character of some polyphenols [42,43,44]. In fact, in a previous study, chia defatted flour showed positive effects on the intestinal microbiota using an in vitro gastrointestinal digestion model (increasing *Lactobacillus* and decreasing *Enterobacteriaceae*) [11]. 

Taken together, all these results confirmed the antioxidant capacity of the supplementation with chia defatted flour. 

## 5. Conclusions

A low-nutritional-quality diet derived from real dietary patterns identified in an urban population of Argentina, high in fructose and with a large proportion of saturated fatty acids, leads to an imbalance in carbohydrate metabolism and general redox state in C57BL/6 male mice. However, supplementing this low-quality diet with 10% chia defatted flour helped restore glucose to normal levels and modulate the endogenous antioxidant system of the organism, resulting in a decrease in the protein oxidation products. This study may prove an important antecedent since, unlike others in which diets with disproportionate compositions of macronutrients are used, only the nutritional quality of the macronutrients was modified, and we also propose the reutilization of an industrial byproduct with positive results on health welfare. 

## Figures and Tables

**Table 1:** Diet composition expressed as g of component per Kg of diet.

Component	C	LNQ	LNQ+C
**Proteins (casein)**	160	180	152.3
**Carbohydrates**			
Cornstarch	400	350	329.35
Sucrose	300	-	-
Fructose	-	350	329.35
**Fiber (wheat bran)**	40	-	-
**Corn oil**	60	-	-
**Palm oil**	-	80	66.4
**Chia defatted flour**	-	-	100
Proteins from chia	-	-	27.70
Fiber from chia			41.30
Lipids from chia			13.60
**Vitamins**			
Choline chloride	1	1	1
Inositol	0.2	0.2	0.2
Folic acid	0.005	0.005	0.005
Vitamin D3 (cholecalciferol)	1.13 × 10^−4^	1.13 × 10^−4^	1.13 × 10^−4^
Vitamin E (α-tocopherol)	0.1	0.1	0.1
Vitamin K3 (menadione)	2.5 × 10^−3^	2.5 × 10^−3^	2.5 × 10^−3^
† Supradyn^®^	5 pills	5 pills	5 pills
**Minerals**			
CaCO_3_	13.15	13.15	13.15
KH_2_PO_4_	5.3	5.3	5.3
FeSO_4_	0.27	0.27	0.27
KCl	2.8	2.8	2.8
NaCl	1.72	1.72	1.72
MgSO_4_	0.17	0.17	0.17
AlKSO_4_	0.042	0.042	0.042

C: Control diet. LNQ: Low-Nutritional-Quality diet. LNQ+C: Low-Nutritional-Quality diet supplemented with 10% of chia defatted flour. † Supradyn^®^ is a dietary supplement based on vitamins, minerals, and trace elements that is commercially produced by Bayer S.A., Buenos Aires, Argentina.

**Table 2:** Body weight and food and water intake.

	Body Weight Gain (%)	Food Intake (g/Week/Animal)	Water Intake (mL/Week/Animal)	Liver Weight (g)	Kidney Weight (g)
**C**	80.56 ± 12.56 ^a^	22.4 ± 0.33 ^b^	26.83 ± 1.64 ^a^	1.1197 ± 0.0250 ^a^	0.2117 ± 0.0217 ^a^
**LNQ**	79.77 ± 15.38 ^a^	20.84 ± 0.62 ^c^	23.07 ± 2.10 ^a^	1.1485 ± 0.1550 ^a^	0.2658 ± 0.0559 ^a^
**LNQ+C**	122.63 ± 12.56 ^a^	24.06 ± 0.45 ^a^	24.97 ± 1.54 ^a^	1.1502 ± 0.0983 ^a^	0.2164 ± 0.0496 ^a^

C: Control diet. LNQ: Low-Nutritional-Quality diet. LNQ+C: Low-Nutritional-Quality diet supplemented with 10% chia defatted flour. The results are expressed as the mean ± SE, and different letters indicate significant differences (*p* < 0.05) between the different diet groups for the same parameter.

**Table 3:** GSH and AOPP concentrations in the liver, kidney, and serum.

	GSH			AOPP	
	Liver	Kidney	Serum	Liver	Kidney
**C**	100.00 ± 13.79 ^ab^	100.00 ± 10.98 ^a^	100.00 ± 8.52 ^a^	100.00 ± 11.82 ^b^	100.00 ± 12.01 ^a^
**LNQ**	114.20 ± 14.15 ^a^	113.06 ± 11.03 ^a^	118.86 ± 8.19 ^a^	112.82 ± 11.85 ^a^	103.95 ± 12.05 ^a^
**LNQ+C**	84.28 ± 13.70 ^b^	102.34 ± 10.83 ^a^	115.44 ± 7.62 ^a^	84.37 ± 11.97 ^b^	103.36 ± 11.97 ^a^

C: Control diet. LNQ: Low-Nutritional-Quality diet. LNQ+C: Low-Nutritional-Quality diet supplemented with 10% chia defatted flour. The results are expressed as the mean ± SE of percentages with respect to the control diet. Different letters indicate significant differences (*p* < 0.05) between the different diet groups for the same parameter in the same tissue.

## Data Availability

The datasets generated for this study are available on request to the corresponding author.

## Supplementary Materials

The following supporting information can be downloaded at: https://www.mdpi.com/article/10.3390/foods13050678/s1. Table S1: Chia defatted flour composition; Table S2: Tentative identification of phenolic compounds in defatted sesame flour using HPLC-DAD-QTOF-MS/MS; Table S3: Polyphenol content of diets quantified by HPLC-MS/MS.
