# Supplementary material for: The Effects of Chia Defatted Flour as a Nutritional Supplement in C57BL/6 Mice Fed a Low-Quality Diet
Source: Foods. 2024 Feb 23;13(5):678. doi: 10.3390/foods13050678 (PMC10930959; doi:10.3390/foods13050678)
Supplement: Supplementary file 1 [file foods-13-00678-s001.zip › foods-2593730-supplementary.pdf]

The Effects of Chia Defatted Flour as a Nutritional Supplement in C57BL/6 Mice Fed a Low-Quality Diet

Agustin Lucini Mas [1,2], Alejandra Mariel Canalis [1,3,4], María Eugenia Pasqualini [3,5], Daniel Alberto Wunderlin [1,2] and María Verónica Baroni [1,2,*]

SUPPLEMENTARY INFORMATION

Table S1: Chia defatted flour composition

Composition	Concentration (%)
Moisture (w.b.)	11.80 ± 0.08
Proteins (d.b)	27.70 ± 0.18
Ash (d.b.)	5.62 ± 0.15
Carbohydrates	41.30 ± 0.54 (dietary fibre)
Lípids (d.b)	13.60 ± 0.28
Palmitic acid (16:0)	7.3 ± 0.2
Stearic acid (18:0)	2.8 ± 0.2
Oleic acid (18:1)	7.4 ± 0.8
Linoleic acid (18:2)	22.0 ± 0.1
Linolenic acid (18:3)	60.5 ± 1.2

w.b= wet basis. d.b= dry basis. Proportion of fatty acids are expressed as % of total lipid

Table S2: Tentative identification of phenolic compounds in defatted sesame flour using HPLC-DAD-QTOF-MS/MS.

N°	Compound	Molecular formula	Rt (min)	[M-H]- Cal.	[M-H]- Exp.	Error (ppm)	(MS/MS)	Compound Family
1	Danshensu	$C_9H_{10}O_5$	11.8	197.0455	197.0450	2.6	179	Hydroxycinnamic acid
2	Caftaric acid	$C_{13}H_{12}O_9$	12.2	311.0409	311.0428	6.2	179	Hydroxycinnamic acid
3	Tryptophan	$C_{11}H_{12}N_2O_2$	12.5	203.0826	203.0816	-4.7	186	Aminoacid
4	Caffeic acid hexoside	$C_{15}H_{18}O_9$	13	341.0878	341.0861	5.0	179	Hydroxycinnamic acid
5	Salvianolic acid I/H	$C_{27}H_{22}O_{12}$	13	537.1038	537.1029	-1.7	339; 295	Hydroxycinnamic acid
6	Fertaric acid	$C_{14}H_{14}O_9$	13.3	325.0565	325.0568	-1.0	193	Hydroxycinnamic acid
7	Salvianolic acid E/B/L	$C_{36}H_{30}O_{16}$	14.2	717.1461	717.1518	7.9	519; 339	Hydroxycinnamic acid
8	Caffeic acid	$C_9H_8O_4$	14.6	179.0350	179.0340	5.5	-	Hydroxycinnamic acid
9	Salviaflaside	$C_{24}H_{26}O_{13}$	17.3	521.1301	521.1378	-14.9	359; 197	Hydroxycinnamic acid
10	Rosmarinic acid	$C_{18}H_{16}O_8$	19.1	359.0772	359.0786	3.7	197; 179	Hydroxycinnamic acid
11	Salvianolic acid C	$C_{26}H_{20}O_{10}$	21.6	491.0984	491.100	-3.3	311; 293	Hydroxycinnamic acid
12	Methyl romarinate	$C_{19}H_{18}O_8$	22.5	373.0929	373.0917	-3.3	179	Hydroxycinnamic acid

Table S3: Polyphenol content of diets quantified by HPLC-MS/MS.

	Compound	Concentration (μg/g)		
		**C**	**LNQ**	**LNQ+C**
1	Danshensu	<LD	<LD	0.92 ± 0.17
2	Caftaric acid	<LD	<LD	0.92 ± 0.17
3	Tryptophan	<LD	<LD	3.26 ± 0.59
4	Caffeic acid hexoside	<LD	<LD	2.31 ± 0.20
5	Salvianolic acid I/H	<LD	<LD	1.12 ± 0.13
6	Fertaric acid	<LD	<LD	20.23 ± 2.56
7	Salvianolic acid E/B/L	<LD	<LD	10.86 ± 3.53
8	Caffeic acid	<LD	<LD	1.17 ± 0.13
9	Salviaflaside	<LD	<LD	241.29 ± 10.04
10	Rosmarinic acid	<LD	<LD	95.59 ± 7.99
11	Salvianolic acid C	<LD	<LD	1.64 ± 0.16
12	Methyl romarinate	<LD	<LD	2.14 ± 0.23
	**∑Polyphenols**	<LD	<LD	383.73 ± 11.74

C: Control diet. LNQ: Low Nutritional Quality diet. LNQ+C: Low Nutritional Quality diet supplemented with 10% of Chia defatted flour. Identification of polyphenol compounds in chia defatted flour was developed in Lucini et al., 2020. <LD, below limit of detection. Compounds 1, 2, 4 and 8 were quantified using caffeic acid as reference compound; compounds 3 was quantified using tryptophan; compounds 5, 7, 9, 10, 11 and 12 using rosmarinic acid and compound 6 using ferulic acid.
